# Evidence That Substantia Nigra Pars Compacta Dopaminergic Neurons Are Selectively Vulnerable to Oxidative Stress Because They Are Highly Metabolically Active

**DOI:** 10.3389/fncel.2022.826193

**Published:** 2022-03-04

**Authors:** Anjie Ni, Carl Ernst

**Affiliations:** Department of Human Genetics, McGill University, Montreal, QC, Canada

**Keywords:** dopamine, midbrain development, FOXA, NR4A2, LMX1

## Abstract

There are 400–500 thousand dopaminergic cells within each side of the human substantia nigra pars compacta (SNpc) making them a minuscule portion of total brain mass. These tiny clusters of cells have an outsized impact on motor output and behavior as seen in disorders such as Parkinson’s disease (PD). SNpc dopaminergic neurons are more vulnerable to oxidative stress compared to other brain cell types, but the reasons for this are not precisely known. Here we provide evidence to support the hypothesis that this selective vulnerability is because SNpc neurons sustain high metabolic rates compared to other neurons. A higher baseline requirement for ATP production may lead to a selective vulnerability to impairments in oxidative phosphorylation (OXPHOS) or genetic insults that impair Complex I of the electron transport chain. We suggest that the energy demands of the unique morphological and electrophysiological properties of SNpc neurons may be one reason these cells produce more ATP than other cells. We further provide evidence to support the hypothesis that transcription factors (TFs) required to drive induction, differentiation, and maintenance of midbrain dopaminergic neural progenitor cells which give rise to terminally differentiated SNpc neurons are uniquely involved in both developmental patterning and metabolism, a dual function unlike other TFs that program neurons in other brain regions. The use of these TFs during induction and differentiation may program ventral midbrain progenitor cells metabolically to higher ATP levels, allowing for the development of those specialized cell processes seen in terminally differentiated cells. This paper provides a cellular and developmental framework for understanding the selective vulnerability of SNpc dopaminergic cells to oxidative stress.

## Introduction

Dopaminergic cells of the ventral midbrain that project to the striatum play an essential role in governing motor behavior in mammals. The small cluster of cells in the substantia nigra pars compacta (SNpc) release the neurotransmitter dopamine to neurons of the striatum which project to the basal ganglia. The basal ganglia is connected to the thalamus and motor cortex which imparts control over motor output. The loss of the majority of SNpc cells is the underlying cause of Parkinson’s disease (PD), a progressive movement disorder characterized by resting tremor, bradykinesia, rigidity and non-motor symptoms such as cognitive decline and sleep disturbance (Hayes, 2019). The causes of PD highlight the critical importance of mitochondria and cellular respiration because toxins that inhibit mitochondrial function or mutations in genes that support mitochondrial function associate with cell loss in animal models and humans (Park et al., 2018).

Inhibitors of the electron transport chain, the group of enzymes in the inner mitochondrial matrix that replenishes adenosine triphosphate (ATP) using oxygen, lead to selective destruction of SNpc dopaminergic cells (Langston et al., 1983; Sayre et al., 1991; Betarbet et al., 2000; Morais et al., 2014), suggesting they are particularly sensitive to inhibition of oxidative phosphorylation (OXPHOS) or reactive oxygen species (ROS) created in this process (Jenner, 1993), collectively termed oxidative stress (Haddad and Nakamura, 2015). This idea is supported by the discovery that many genetic mutations associated with PD appear to be associated in some way with mitochondrial dynamics or oxidative stress (e.g., *PARK7*, *PARKIN*, and *PINK1*) (Shen and Cookson, 2004). Furthermore, genetic insults involving other energy pathways also appear to be basal ganglia-related in that they can affect motor function; examples include deficiency of glucose transporter *SLC2A1* (Leen et al., 2010) (*OMIM 606777*) or *GCH1* (Trender-Gerhard et al., 2009) (*OMIM 128230*). The links between oxidative stress and basal ganglia-related movement disorders lead to the fundamental question of what drives the underlying cause of disease. There are many potential explanations for this, but here we will evaluate the evidence that SNpc dopaminergic cells have higher ATP requirements than other brain cell types making them more susceptible to genetic insults or toxins which impair OXPHOS. The motivation for this piece came from our recent serendipitous discovery showing that the dopaminergic midbrain progenitors were operating at full OXPHOS capacity compared to isogenic glutamatergic forebrain progenitor cells (Bell et al., 2021). While this was not the primary concern of that paper, the effect size in healthy, unbranched, and non-dopamine producing cells was striking. Might SNpc cells then be programmed to high energy states? We wanted to pursue this idea more in depth, hence the current article. Here, we will argue that selective vulnerability is because SNpc neurons operate at a higher baseline levels of ATP synthesis/oxygen consumption both because they are intrinsically programmed that way, and must sustain highly energy consuming processes due to particular cell properties. While this idea is not mutually exclusive from other ideas such as the selective vulnerability to ROS in SNpc, a formal review of this idea is warranted.

We first define the concept of selective vulnerability in SNpc DA cells, then review evidence in two parts. In the first part we focus on morphological and electrophysiological properties of terminally differentiated SNpc neurons. We review findings showing that SNpc neurons are more vulnerable to OXPHOS insults than other dopaminergic cell types, then discuss the morphological and electrophysiological properties of SNpc dopaminergic neurons that contribute to increasing ATP demands. In the second part, we focus on transcription factors (TFs) required for the development of midbrain SNpc progenitor cells. We review the role of these TFs in both SNpc development and metabolism, and suggest that the dual feature of these TFs is particular to SNpc cells. SNpc cells may be programmed to a higher energetic state before the differentiation of morphological and electrophysiological cell properties emerges. This might suggest that developmental programming of midbrain progenitors allows for the terminal differentiation of highly ATP consuming processes, rather than the other way around.

## Dopaminergic Cells of the Substantia Nigra Pars Compacta and the Concept of Selective Vulnerability

Dopamine is part of the catecholamine family of monoamines, defined by the presence of an amine and a catechol group, derived from phenylalanine or dietary tyrosine. It is synthesized in several areas in mammals including chromaffin cells of the adrenal medulla and in at least 11 clusters in brain from the olfactory bulb to the midbrain (Felten and Sladek, 1983). One such cluster resides in the ventral tegmental area (VTA) of the midbrain (A10 area in mouse), where dopaminergic neurons project to the nucleus accumbens. Another such cluster lies in the SNpc (A9 in mouse), which is adjacent to the VTA and comprises neurons that project to the striatum. The million or so cells that make up the bilateral substantia nigra have an outsize impact on functional output. Destruction of tens of millions of cortical cells after a stroke for example, can lead to hemi-paralysis which in many cases can be partially recoverable (Murphy and Corbett, 2009), while loss of a majority of the dopamine producing cells of the midbrain SNpc can lead to significant motor impairment. While dopamine replacement therapy can alleviate some PD symptoms in most people, cell destruction and degeneration are not slowed.

Selective vulnerability refers to the idea that a given insult affects a given cell type more negatively than another cell type. For example, the expanded CAG repeat in the *HUNTINGTIN* gene causes Huntington’s disease which primarily affects medium spiny neurons of the striatum (Ehrlich, 2012). Similarly, there are germline mutations that lead to very specific cancer types, despite expression of a mutated gene in all or most areas of the body; one example is that of *RUNX1* germline mutations predisposing to blood cancer (Avagyan and Brown, 2021). The different molecules that define a cell state are likely also the same factors that can lead to selective vulnerability. Since tissue types are different by definition, it becomes easier to understand how some cell types can be vulnerable to specific insults (whether genetic or environmental) than others. In the case of SNpc cells, they are much more vulnerable to inhibitors of OXPHOS (reviewed below). The depletion of ATP might force cells into cell death programs if they cannot sustain their basic energy needs (Kushnareva and Newmeyer, 2010).

## Part A: Morphological and Electrophysiological Properties of Terminally Differentiated SNpc Dopaminergic Cells

### Substantia Nigra Pars Compacta Dopaminergic Cells Are More Vulnerable to Electron Transport Inhibitors Than Other Dopaminergic Cell Types

Mitochondria provide about 93% of the ATP in the brain (Sokoloff, 1960) and most of the ATP required for neuronal activity (Hall et al., 2012; Harris et al., 2012) making OXPHOS critical for global brain function. In rodent studies testing exposure to MPP^+^ (5–10 μM) or rotenone (25–50 nM), both of which inhibit mitochondrial complex 1, loss of dopaminergic neurons in the SNpc is significantly higher than in dopaminergic neurons from the VTA, the midbrain area adjacent to the SNpc (Pacelli et al., 2015; Jaumotte et al., 2016), a result supported by *in vitro* human findings (Oosterveen et al., 2021). The VTA is a favored comparator region (Chung et al., 2005) because these cells are dopamine-producing, they are adjacent to SNpc (so presumably follow at least similar developmental trajectories), but are often preserved in PD (Hirsch et al., 1988; Maingay et al., 2006). Under low doses of 6-OHDA, a toxin that also impairs mitochondrial complex 1 and a commonly used approach to model PD in animals, SNpc dopaminergic neurons show a loss of 40% while VTA dopaminergic neurons do not show significant loss (Giguere et al., 2019; Tanguay et al., 2021). SNpc neurons seem to be more susceptible to impairment of OXPHOS compared to dopaminergic cells in the VTA (Subramaniam and Chesselet, 2013). A complete comparison of VTA and SNpc cells in the respect to selective vulnerability has been described (Brichta and Greengard, 2014).

### Substantia Nigra Pars Compacta Dopaminergic Cells Have More Energy Consuming Synaptic Contacts Compared to Other Dopaminergic Cell Types

A single SNpc neuron has a very large number of projections into the striatum (Anden et al., 1966), and it has been possible to trace these large structures in a dissected brain over long distances and branch points. Matsuda et al. (2009) showed that one single SNpc neuron can form connections with, on average, 2.7 ± 1.5% of striatal neurons (Matsuda et al., 2009; Bolam and Pissadaki, 2012). Comparing subtypes of dopaminergic cells in rat shows that SNpc TH-positive neurons are 69 and 326% larger than that of VTA and OB TH-positive neurons (Pacelli et al., 2015) and that a single SNpc neuron forms from 100,00 to 250,000 connections in the rat striatum (Bolam and Pissadaki, 2012) whereas a single VTA DA neuron makes 12,000 to 30,000 connections. Based on these numbers and the known differences between rat and human brains, it is estimated that one single human SNpc neuron could have 1 million to 2.5 million striatal synaptic sites (Bolam and Pissadaki, 2012). This work is supported by diffusion tensor imaging studies from human brain suggesting significantly more connections from SN than VTA (Kwon and Jang, 2014). SNpc DA neurons are thus not only highly branched, but they are also highly active given the number of synaptic sites.

Why might extensive branching and connections cause increased energy burden, as defined by ATP utilization? The number of ATP molecules required to restore membrane potential after the propagation of an action potential exponentially increases as a function of the size and complexity of arborization (Pissadaki and Bolam, 2013). The high energy cost associated with sustaining such activity is known to translate into higher mitochondrial biogenesis, density, and respiration in cultured primary neurons (Pacelli et al., 2015). Compared to VTA neurons, SNpc neurons show significantly higher levels of PGC-1α, a regulator of mitochondrial biogenesis, and higher mitoDSRed signal, a proxy for mitochondrial network expression. Furthermore, basal oxygen consumption rates (OCR) of SNpc neurons are threefold higher than that of VTA or OB neurons (Pacelli et al., 2015). SNpc neurons do not significantly differ from the two other DA cell types in terms of maximal OCR, so the results suggest that SNpc DA neurons function at maximal capacity under baseline conditions whereas VTA and OB dopaminergic cell types have the potential to achieve this high energy state only if required by metabolic pressures.

### Electrophysiological Activity of Substantia Nigra Pars Compacta Neurons Require More ATP Than Other Dopaminergic Cell Types

Substantia nigra pars compacta DA neurons have unique electrophysiological properties. They are autonomous pacemakers (Clark and Chiodo, 1988; Grace and Onn, 1989; Hyland et al., 2002), transiently firing broad and slow action potentials in addition to burst firing, in a process that is dependent on Ca^2 +^ influx (Puopolo et al., 2007). SNpc neurons not only have to ensure that their electrochemical environment allows firing thresholds to be reached, but must also do so with high frequency, to maintain tonic rhythm. Ca^2 +^ entry *via* Ca^2 +^ ATPase channels is essential in sustaining such electrochemical gradients, but is relatively expensive in energy as the channels consume one molecule of ATP to pump one molecule of Ca^2 +^ (Surmeier et al., 2012). In comparison, one molecule of ATP can be used to pump three Na^+^ ions and two K^+^ ions via Na^+^/K^+^ -ATPase channels, which are required to maintain electrochemical gradients for action potentials in, for example, cortical pyramidal neurons. Further, SNpc neurons’ activity involves L-type Cav1.3 Ca^2 +^ channels (Chan et al., 2007; Guzman et al., 2009), a type of channel that is rarely opened or lowly expressed in other brain regions and that opens at steep subthreshold membrane potentials (Wilson and Callaway, 2000; Puopolo et al., 2007). This causes large amounts of Ca^2 +^ to flow in the SNpc neurons, which increases the cells’ metabolic load (Guzman et al., 2010; Goldberg et al., 2012). Support for this idea can be seen by blocking L-type Cav1.3 Ca^2 +^ channels in rodent SNpc brain slices which leads to decreased oxygen consumption and lower levels of ATP (Guzman et al., 2010).

The influx/efflux of calcium is costly, but required in SNpc cells. In VTA DA neurons, Ca^2 +^ can be sequestered by Ca^2 +^ -buffering proteins which are not ATP-dependent and thus “energetically cheap” to maintain (Surmeier et al., 2012), so SNpc cells have a particular need for readily moveable Ca^2 +^ across the membrane and cannot benefit as much from cheap Ca^2 +^ buffering used in VTA neurons. SNpc neurons do buffer calcium but this is low relative to their Ca^2 +^ influx during pacemaking activity (Foehring et al., 2009), so SNpc cells may rely on a more costly mechanism to regulate Ca^2 +^ levels. An example of such mechanism is Ca^2 +^ sequestration by the ER network, which also requires ATP-dependent transporters and mitochondrial Ca^2 +^ uniporter [reviewed in (Surmeier et al., 2012)].

## Part B: Developmental Programming of Ventral Midbrain DA Cells

### Substantia Nigra Pars Compacta Cells Are Induced via a Series of Transcription Factors With Known Roles in Metabolism

Several TFs are required to induce, differentiate, or maintain SNpc neurons throughout development, and many of these are not specific to SNpc but rather are specific to dorsal-ventral or anterior-posterior patterning. What defines an SNpc neuron is not any one TF but rather the combination of TFs at a given time and 3D position on these axes. In this way, an organism uses the same TFs, in for example, ventral patterning, such as LMX1A to contribute to specification of both motor neurons and SNpc neurons. Some of the TFs that contribute to SNpc fate specification are very well known for their roles in metabolism, a function presumably unrelated to their developmental patterning roles, although it is possible that their metabolic function is in fact patterning these cells as well. Here, we describe several TFs important in SNpc neurons at different developmental stages and described both their role in SNpc fate specification and metabolism. We note that studies investigating the metabolic roles of genes described here are mostly conducted in liver, kidney, pancreas, and muscle. Studies showing the direct relationship between midbrain patterning TFs and energy expenditure/storage in the brain have not yet, to our knowledge, been investigated.

Recently, we found strong evidence that undifferentiated but committed cells that can give rise to SNpc cells have much higher baseline OXPHOS and ATP levels than forebrain cells that were developmentally comparable and isogenic (Bell et al., 2021). This suggested to us that SNpc cells may be programmed to a high energy state prior to the differentiation of morphological or electrophysiological properties characteristic of SNpc neurons described in the first section of this paper. If this were true, it may imply two things: (1) That the TFs that induce cells to become committed progenitors of SNpc fate are programming the cells very early on to high ATP consumption and high OXPHOS levels, and (2) The high ATP-requiring processes of SNpc cells may have been able to develop only because of this programming step; that is, they are a consequence of the developmental programming, rather than high ATP consumption being a consequence of particular cell morphology and electrophysiological characteristics.

LMX1A/B, NR4A2, EN1/2, OTX1/2, CORIN, PITX3, and FOXA1/2 are probably the best described TFs that have a role in SNpc fate specification (Asgrimsdottir and Arenas, 2020), and some of these have a role in metabolism, which are the TFs we focus on here. Our purpose in this section is to highlight that ventral midbrain programming uses TFs that have both a metabolic role and a cell patterning role. While we cannot rule out that other TFs that drive cell fate for other neuronal cell types also have roles in metabolism that have yet to be discovered (e.g., PAX6 or FOXG1 in forebrain), the well-known role of the ventral midbrain neuronal factors in metabolism is possibly unique to TFs that drive SNpc neuron development.

**FOXA1 and FOXA2** are part of the FoxA subfamily of the forkhead/winged-helix family. They are key regulators of neural tube patterning (Monaghan et al., 1993; Sasaki and Hogan, 1993) and are expressed both in ventral midbrain progenitors early in development (E8.5 onward in mouse) (Ang et al., 1993) and in terminally differentiated neurons (Ferri et al., 2007), so may have both an inductive and maintenance function. Homozygous deletion of *Foxa2* in mice results in the absence of a notochord, and thus the absence of a floor plate and abnormality in the dorsal-ventral patterning of the developing neural tube (Ang and Rossant, 1994), but brain specific knock-outs have better refined their role in the ventral midbrain. Specifically, Foxa1/2 regulate the extent of neurogenesis and thus the final number of SNpc neurons, as well as the activation of other important TFs in midbrain and described here (Ferri et al., 2007). In later stages of development, brain specific knock-out of both *Foxa1/2* results in less tyrosine hydroxylase and less burst firing specifically in SNpc neurons (Pristera et al., 2015).

With respect to metabolic function, Foxa1 and Foxa2 are required for regulating glucose homeostasis (Kaestner et al., 1999; Lantz et al., 2004; Friedman and Kaestner, 2006). *Foxa1*-null mice, despite being fed, show glucose levels similar to that of starved animals, which results in death of the animals due to hypoglycemia (Kaestner et al., 1999). This is due to inefficient glucagon signaling likely due to poor *Gcg* gene expression in response to hypoglycemic conditions, a result supported by *in vitro* studies (Philippe et al., 1994). Under fasting conditions, mice hepatocytes deficient in *Foxa2* show significantly lower cAMP and glucocorticoid signaling and blunted efficiency of a cAMP target protein (CREB) binding to its target sites (Zhang et al., 2005). In pancreatic beta cells, cell-specific ablation of *Foxa2* results in the complete loss of K-ATP channels, which normally couple glucose metabolism with insulin secretion (Lantz et al., 2004). Specifically, *Foxa2* mutant mice (Sund et al., 2001; Lantz et al., 2004) show significant reductions of both K-ATP channel subunits, *Abcc8* and *Kcnj11* (Reis and Velho, 2002; Vaxillaire et al., 2004), genes that are also markers of midbrain dopaminergic cells (Liss et al., 1999; Schiemann et al., 2012). FOXA1/2 may program both pancreatic beta cells and ventral midbrain NPCs and use the same molecular tools (e.g., K-ATP subunit genes) in different tissue types to achieve different ends (presumably insulin secretion and regulation of calcium transients, respectively). Finally, Foxa2 is regulated by insulin itself (Wolfrum et al., 2003) and several single nucleotide polymorphisms (SNPs) in the gene are significantly associated with different metabolic indicators, such as fasting blood glucose levels [e.g., rs6048205 (Manning et al., 2012; Wojcik et al., 2019; Sinnott-Armstrong et al., 2021), rs3833331 (Chen et al., 2021; Sakaue et al., 2021), rs1209523 (Xing et al., 2010; Wojcik et al., 2019), rs72470563 (Chung et al., 2021), rs1337918 (Chen et al., 2021)].

**NR4A2** is a nuclear receptor that belongs to the nuclear receptor subfamily 4 group A (NR4A) and is also known as NURR1. Knockout of *Nurr1* in mice results in complete loss of midbrain dopaminergic cells, implying that Nurr1 is required for induction of ventral midbrain cell fate (Zetterstrom et al., 1997) and the survival of SNpc neurons (Zetterstrom et al., 1997; Saucedo-Cardenas et al., 1998). In metabolism, *Nr4a2* is a key regulator in glucose metabolism, lipid metabolism, and the TCA cycle [reviewed in Herring et al. (2019)]. In isolated mouse primary hepatocytes, gluconeogenesis stimulates the expression of *Nurr1*, and fasting mice show increased *Nr4a2* expression in extracted livers. NR4A family members can bind the promoters of the gluconeogenesis genes and a glucose transporter gene *Glut2* (Pei et al., 2006). Outside of the liver, Nr4a2 is upregulated in skeletal muscle following endurance exercise (Mahoney et al., 2005; Catoire et al., 2012) and has a critical role in glucose uptake (Amoasii et al., 2016, 2019).

**LIMX1A/B** are part of the LIM homeodomain family of genes and knockout in mice strongly links them the SNpc dopaminergic cell development (Yan et al., 2011). During brain development, at around embryonic day 9.5 in mice, *Lmx1a* is expressed in the roof plate of the neural tube and is responsible for neural tube specification (Millonig et al., 2000; Chizhikov and Millen, 2004). Lmx1a specifically mediates the differentiation of floor plate dopaminergic progenitors (Deng et al., 2011), whereas Lmx1b is involved in the establishment of the midbrain-hindbrain boundary and mediates the differentiation of lateral, non-floor plate, dopaminergic progenitors (Guo et al., 2007; Deng et al., 2011). Recent evidence shows that Lmx1b is required for the autophagic-lysosomal pathway and neuroprotection of post-mitotic dopaminergic neurons (Laguna et al., 2015; Doucet-Beaupre et al., 2016).

In pancreatic beta islet cells, Lmx1a regulates the expression of insulin *via* a synergistic interaction with the basic helix-loop-helix protein E47/Pan-1 at the promoter of the insulin gene (German et al., 1992, 1994). Lmx1b might also regulate genes that respond to a diet switch (from normal food diet to a restricted food diet) in drosophila (Whitaker et al., 2014). In humans, several polymorphisms in *LMX1B* have been associated with metabolic diseases. The polymorphism rs10733682 is associated with BMI (Locke et al., 2015) and the AA genotype of rs10733682 interacts with intake of total energy, fat, and carbohydrate in determining risk of developing elevated triglycerides levels, which is associated with higher obesity incidence (Zhu et al., 2020). Numerous other *LMX1B* variants have been associated with BMI or fat body mass in genome-wide association studies, such as rs867559 (Speliotes et al., 2010), rs7027304 (Tachmazidou et al., 2017; Sakaue et al., 2021), rs3829849 (Costa-Urrutia et al., 2020), rs2235056 (Akiyama et al., 2017), rs1336980 (Vogelezang et al., 2020), rs7030609 and rs2275241 (Richardson et al., 2020).

## Conclusion

We provide several lines of evidence that SNpc cells have higher metabolic activity than other cell types including other dopaminergic cell types outside the SNpc. In part A of this work, we looked at the cellular properties of these cells. The evidence reviewed might suggest that SNpc cells require more ATP and thus more OXPHOS to sustain and maintain these particular functions than other cells and so are selectively vulnerable to genetic or metabolic insults, such as mutations in genes that support mitophagy or chemicals that interfere with Complex I of the electron transport chain. We do not necessarily consider this idea exclusive from other hypotheses of SNpc selective vulnerability, namely the creation of ROS from OXPHOS or the oxidation of dopamine itself.

In Part B, we proposed a new idea for the association between SNpc cells and a heightened energy state. We suggest that SNpc neurons may be programmed to this state prior to the development of potentially energy intensive cellular activities. Committed ventral midbrain progenitor cells are unbranched, have no synaptic contact, and do not fire action potentials, yet we previously found that OXPHOS capacity is much higher than comparator forebrain progenitor cells (Bell et al., 2021). This could be direct evidence that high levels of OXPHOS are already present in progenitor cells prior to the development of electrophysiological or cellular properties. We suggest that this state may come about due to the unique assortment of TFs required to induce SNpc development. Specifically, we highlight TFs that are also known actors in metabolic programming with essential roles in insulin and glucose maintenance. It may be that the metabolic role of these TFs is in fact the programming step, and it is not that these TFs have independent and dual roles in metabolism and SNpc development.

## Author Contributions

Both authors wrote the manuscript, contributed to the article and approved the submitted version.

## Conflict of Interest

The authors declare that the research was conducted in the absence of any commercial or financial relationships that could be construed as a potential conflict of interest.

## Publisher’s Note

All claims expressed in this article are solely those of the authors and do not necessarily represent those of their affiliated organizations, or those of the publisher, the editors and the reviewers. Any product that may be evaluated in this article, or claim that may be made by its manufacturer, is not guaranteed or endorsed by the publisher.
